# Artificial and natural silk materials have high mechanical property variability regardless of sample size

**DOI:** 10.1038/s41598-022-07212-5

**Published:** 2022-03-03

**Authors:** Gabriele Greco, Hamideh Mirbaha, Benjamin Schmuck, Anna Rising, Nicola M. Pugno

**Affiliations:** 1 Laboratory for Bioinspired, Bionic, Nano, Meta, Materials and Mechanics, Department of Civil, Environmental and Mechanical Engineering, University of Trento, Via Mesiano, 77, 38123 Trento, Italy; 2 Department of Anatomy, Physiology and Biochemistry, Swedish University of Agricultural Sciences, Uppsala, Sweden; 3 Department of Biosciences and Nutrition, Karolinska Institutet, Neo, 141 86 Huddinge, Sweden; 4 School of Engineering and Materials Science, Queen Mary University of London, Mile End Road, London, E1 4NS UK

**Keywords:** Biomaterials, Soft materials, Structural materials

## Abstract

Silk fibres attract great interest in materials science for their biological and mechanical properties. Hitherto, the mechanical properties of the silk fibres have been explored mainly by tensile tests, which provide information on their strength, Young’s modulus, strain at break and toughness modulus. Several hypotheses have been based on these data, but the intrinsic and often overlooked variability of natural and artificial silk fibres makes it challenging to identify trends and correlations. In this work, we determined the mechanical properties of *Bombyx mori* cocoon and degummed silk, native spider silk, and artificial spider silk, and compared them with classical commercial carbon fibres using large sample sizes (from 10 to 100 fibres, in total 200 specimens per fibre type). The results confirm a substantial variability of the mechanical properties of silk fibres compared to commercial carbon fibres, as the relative standard deviation for strength and strain at break is 10–50%. Moreover, the variability does not decrease significantly when the number of tested fibres is increased, which was surprising considering the low variability frequently reported for silk fibres in the literature. Based on this, we prove that tensile testing of 10 fibres per type is representative of a silk fibre population. Finally, we show that the ideal shape of the stress–strain curve for spider silk, characterized by a pronounced exponential stiffening regime, occurs in only 25% of all tested spider silk fibres.

## Introduction

**Figure 1:**
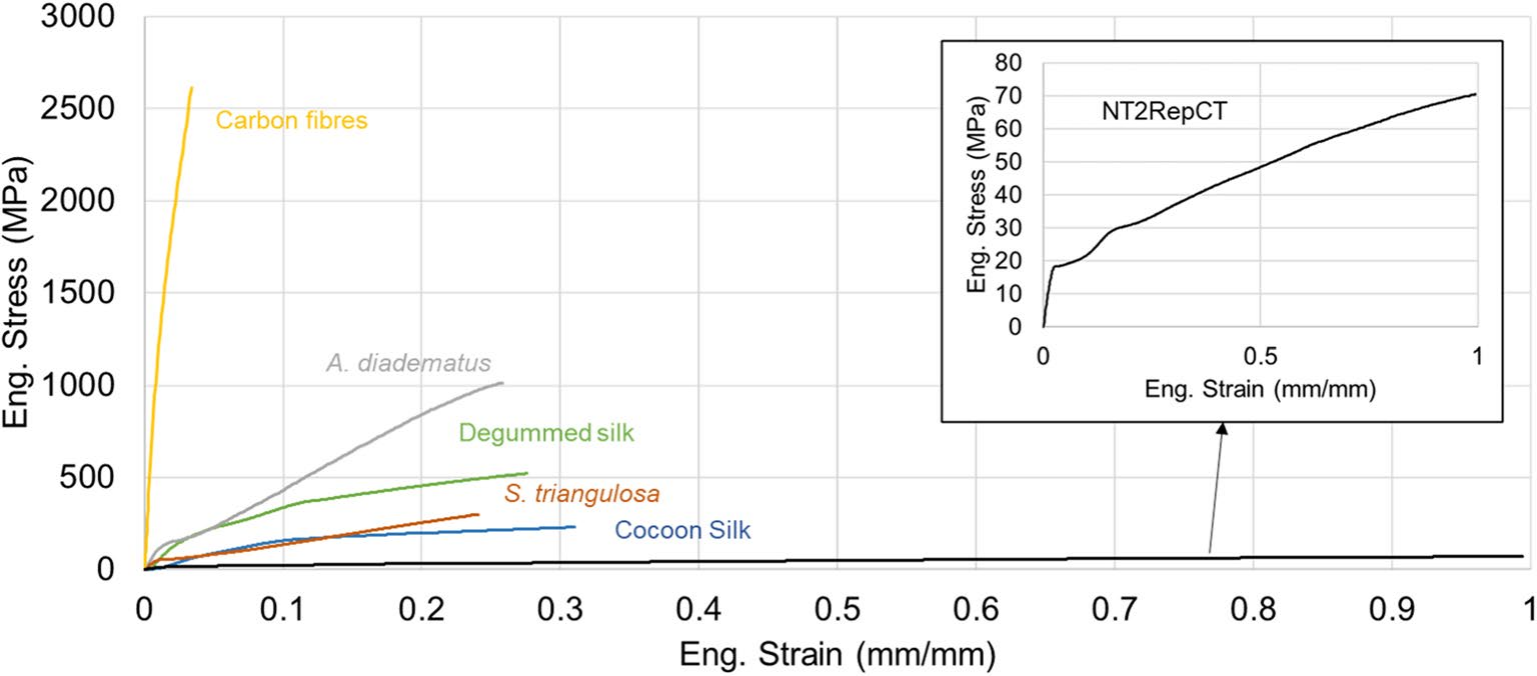
Representative Stress–Strain curves of different fibres: commercial carbon fibres, A. diadematus major ampullate silk, Bombyx mori degummed and raw silk, S. triangulosa major ampullate silk, and the NT2RepCT artificial spider silk.

Among fibrous materials, natural silks (spun by spiders and silkworms) are known for their remarkable biological, structural, and mechanical properties^1–3^ (see some examples in Fig. 1). In particular, the strength of silk matches the strength of high-strength steels, while the extensibility of silk outperforms many polymeric materials^4–7^. In combination, these two characteristics make silk one of the toughest natural materials there is^8^, and as such, silk is potentially suitable as a high-performance textile^9^ and, because of its biocompatibility, also for biomedical applications^10–13^.

Spiders and silkworms use their silk for their biological needs, the former for hunting^3,14^ and the latter for producing cocoons^4,15^, which do not require a large amount of this proteinaceous material. Moreover, spiders have a cannibalistic nature^16^, and for these reasons, it is extremely challenging to employ these animals to upscale the production of silk^17^, which makes the amount of available natural silks strictly limited. In this context, a positive recent advancement is the synthesis of the highly soluble minispidroin NT2RepCT^18^, which made efficient biomimetic spinning of artificial spider silk fibres possible. Moreover, NT2RepCT can be produced in extremely high yields in bioreactors, which opens the gateway for economically and environmentally friendly large scale production of artificial silk fibres with good mechanical properties compared to other artificial spider silks (Fig. 1)^18–22^.

When studying the mechanical properties of the NT2RepCT fibres, we discovered a large inherent fibre to fibre variability both within one set of fibre samples, and between different experimental studies^22–24^. Such variability can also be found in natural silk fibres^1,25–28^. The main reasons for the observed variability can be summarized as follows. First, humidity irreversibly plasticizes native spider silk (if unrestrained)^29,30^, regenerated *Bombyx mori* silk^31^, and artificial spider silk^24,32^, which means that humidity influences the tensile test results. In this respect, not only the environmental conditions at the time of measurement are relevant, but also the conditions the fibres experience after spinning. Second, since silk is a viscoelastic material^33^, forced silking procedures and spinning conditions affect the silk tensional states, the secondary structure content of the fibres, and thus their mechanical properties^34,35^. The same is true for artificial silk fibres as well as silk fibroin fibres, where the spinning protocols or the degumming process affect the mechanical properties of the fibres^23,36,37^. Third, a major cause for the variability may be found in the fact that the calculation of the stress is dependent on a precise measurement of the fibre cross-sectional area. To compute the strength, the load is divided by the cross-sectional area of the fibre, commonly obtained from its diameter. Considering that the shape of the fibre cross section may substantially deviate from a perfect circle and it can be not homogeneous along the length of the fibre, this procedure can lead to an over or underestimation of the real strength and Young's modulus and increase the variability of the results^38^.

To present a specific mechanical property (e.g. Young’s modulus, strain at break, strength, or toughness modulus) the average (*μ*_*s*_) and the standard deviation (*σ*_*s*_) of the sample (whose size, i.e. the number of tested specimen, is *n*) are reported, which should be representative of the ones of the population, *μ* and *σ* respectively. Thus, the sample’s mechanical property should be expressed as *μ*_*s*_ ± *σ*_*s*_. Ideally, the higher the *n* the closer *μ*_*s*_ and *σ*_*s*_ are to *μ* and *σ* respectively, and thus the sample could be considered a good representation of the population. The parameters *μ*_*s*_ and *σ*_*s*_ depend on the type of silk that is tested, but a relatively low standard variation (< 10% of the average) is commonly declared, despite that the sample size is relatively small (≤ 10)^8,30,39,40^. Given that the mechanical properties of silk materials are inherently variable due to the previously mentioned factors, we believe that a more profound investigation on the variability of the mechanical properties of silk fibres should be performed, exploring different sample sizes.

Molecular dynamics simulations have shown that spider major ampullate silk fibres possess a particular constitutive law, i.e. stress–strain behaviour^41–43^. This behaviour is characterized by an initial linear regime before the yielding point, followed by a second nonlinear elastic stiffening regime that is followed by yet another linear regime prior to failure. The nonlinear stiffening regime is recognised to be a peculiarity of spider major ampullate silk, despite it can also be found in non-mulberry silkworm silk^44,45^, and as one of the reasons for its extraordinary efficiency in reducing the damage after impact and preserving robustness when it used in the structures built by the animal (e.g. orb webs^41,46,47^). The spider silk stiffening regime is defined by the following nonlinear stress–strain relation:

1 $$s = k*\ln^{\alpha } \left( {\epsilon + 1} \right)$$

where *s* is the stress, *k* is a constant, *ε* the strain, and *α* > *1* is the parameter used to define the nonlinearity and the stiffening. In simulations, this parameter is an input and it is commonly taken >  > 1^41,46,47^. Even though a particular shape of the stress–strain curve is commonly discovered in ideal simulations, it is seldomly found in real experiments^3,8,12,25–27,48,49^. For instance, the *α* parameter was recently measured for several spider silk fibres, obtaining values between 1.2 and 1.5^14^. This suggests that the strong nonlinearity assumed for spider major ampullate silk may only be found in particular species (e.g. orb weavers), and adds another layer of variability that should be explored.

Herein to better understand the variability of silk materials, we collected several mechanical property datasets for different fibrous materials obtained from different samples sizes (namely 10, 20, 30, 40, and 100). More specifically, the variability of the mechanical properties of artificial spider silk (spun from NT2RepCT), native spider silk, *Bombyx mori* cocoon and degummed silk, as well as commercial carbon fibres for comparison were investigated, including the qualitative shape of the stress–strain curves. In summary, this work shows that the variability of the mechanical properties of artificial and native silk fibres is high, regardless of the number of tested specimens, highlighting the importance of reporting all the data without selection and demonstrating that 10 fibres may be sufficient to represent the population. Moreover, the results show that the nonlinear stiffening behaviour of spider major ampullate silk is commonly defined with *α* < 1.5 (75% of the cases) and does not depend on spider species (orb-weaver or not). This could be helpful for future simulations works in which the constitutive law of major ampullate silk is required.

## Results and discussion

The mechanical properties of the different types of fibres were obtained utilizing tensile tests. For each type of fibre 200 specimens were tested and divided into samples of 10, 20, 30, 40, and 100 respectively. For each sample, the mean values and standard deviation of strain at break, Young’s modulus, eng. strength and toughness modulus were obtained and plotted against the sample’s size. The types of materials that were tested were carbon fibres, artificial spider silk (spun from NT2RepCT^18^), native major ampullate spider silk (*Steatoda triangulosa*), *B. mori* cocoon and degummed silk.

**Figure 2:**
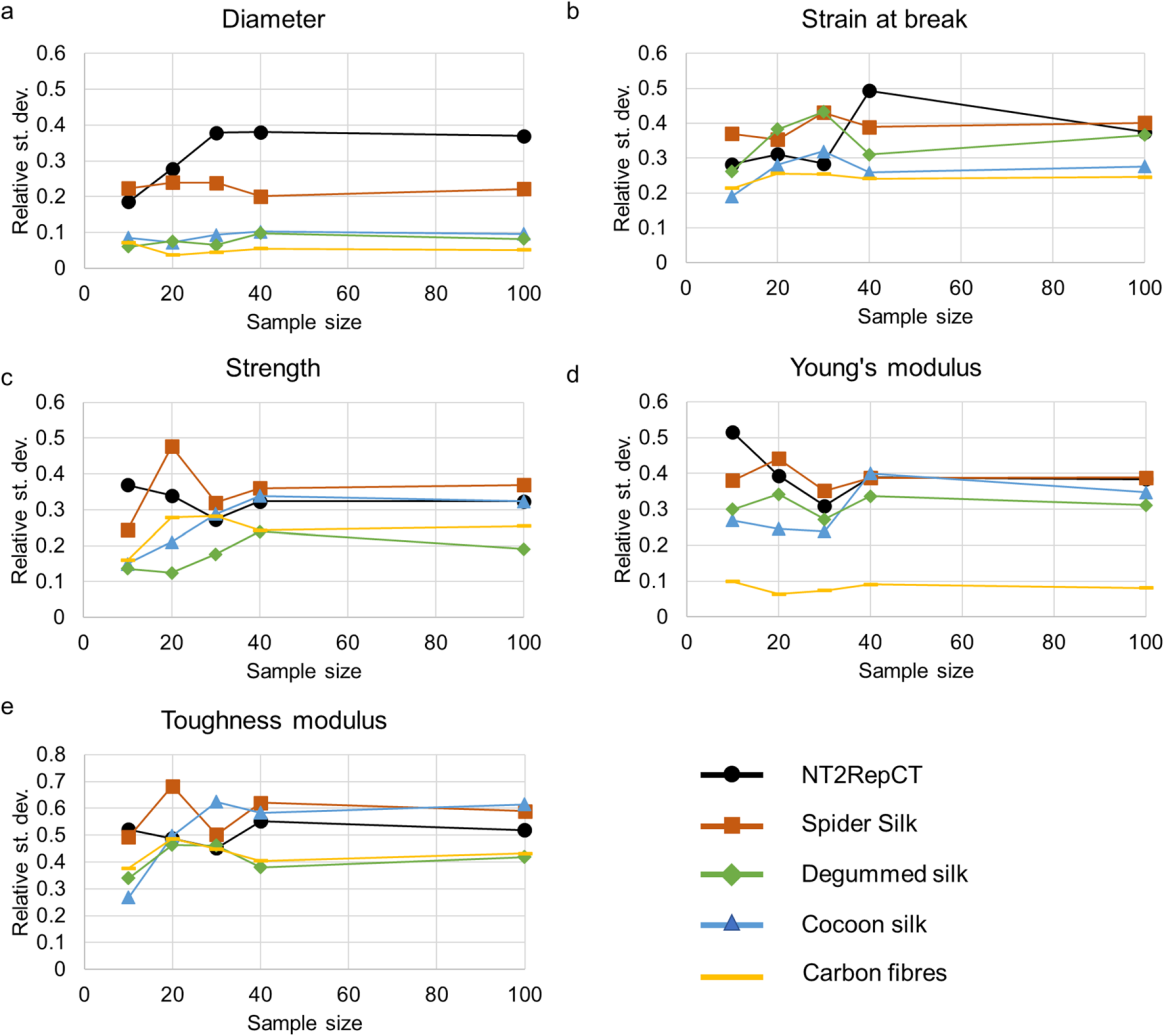
Relative standard deviations of the different mechanical properties (**a**) diameter, (**b**) strain at break, (**c**) strength, (**d**) Young’s modulus, and (**e**) toughness modulus for the different considered fibres.

The diameters of the analysed fibres were different between the fibre types (Fig. S1), and spanned from 2.5 μm (for spider silk) to 30 μm (for *B. mori* cocoon silk). However, for the same fibre type, there was no difference in the average diameter between the sample groups (10, 20, 30, 40, and 100). With respect to the variability of the diameter, the highest standard deviation (relative to the average) was found for artificial spider silk (up to 40%, Fig. S1a), regardless of the sample size (Fig. 2a). These high values likely originate from the previously reported non-homogeneous dual fibre shape with a longitudinal groove^22–24,50^. This issue is often neglected even though it is a common problem in artificial silk and non-silk fibre spinning^40,51,52^. The non-uniform shape will affect the measurement of the diameter and thus contribute significantly to the inherent variability within the sample, as it prevents a correct calculation of the applied stress.

Slightly lower variability in diameter has been observed for native spider silk (20–25%, Fig. S1b), also regardless of the sample size. This could be due to the spinning conditions and the interaction with the animal during the milking procedure^34^. Indeed, it is well known that spiders, even if they are forced to spin, produce silk lines with a certain variability in diameter (up to 30%)^49,53,54^. As expected, the variability in diameter of the other types of fibres, was consistently lower, regardless of the sample size (Fig. S1c-e). This could be due to the industrial spinning protocols for carbon fibres, which require high standardization, and to uniform spinning behaviour of silkworms^55^.

The strain at break and the strength, expressing the degree at which a fibre can sustain elastic and plastic deformation prior to fracture, were very variable for all the tested fibres types, and were independent of the sample size (Figs. 2b-c and S2-3). Again, the lowest and highest variability was observed for carbon fibres and artificial spider silk, respectively. One of the causes of silk variability could be the presence of weak segments in the fibres induced by the spinning behaviour of the spider, artificial spinning procedure or defects introduced during fibre spinning and mounting^38,57–60^. Such segments have been proposed to exist both in artificial silk fibres^56^ and also for non-mulberry silkworm silk that presents lower variability and a more consistent stress–strain profile^45^. In this sense, the silk sample exhibiting the lowest variability was degummed silk. We hypothesise that this lower variability may be caused by the degumming process^36^ that consists in boiling the fibres for a prolonged time in a solution with water and sodium carbonate, which may affect the secondary structure content and intermolecular contacts in the fibre and its tensile properties. The impact of water in silk secondary structure has been deeply investigated with the phenomenon called supercontraction^24,61–63^, which leads to a re-organization, via disruption and creation of hydrogen bonds, of fibre’s molecular structure and drastically changes its mechanical properties, eliminating residual stresses (the so-called ground state) if the fibres are dried^31,39,64,65^. Interestingly, it should be noted that immerging silk fibres in a polar solvent is a technique that has been used in the past to homogenize the properties of spider silk^66^.

In terms of Young’s modulus, the lowest variability (regardless of sample size) was observed for carbon fibres (Figs. 2d and S4). The pure elastic behaviour of carbon fibres may be an explanation for this low variability compared to silk fibres, which are strongly viscoelastic and whose Young’s modulus is calculated in the initial linear regime of the stress–strain curve, strongly affected by pre-stress^33,67^.

For the toughness modulus, the variability was always high (> 25%) regardless of the sample size (Figs. 2e and S5), for all the samples tested. In this case, such variability may be induced by error propagation ^38,68^, since toughness modulus is derived by summing the multiplication between stress and the increments of the strain.

In summary, as elucidated by Fig. 2, the variability of silk fibres’ mechanical properties is high (10% to 70%), regardless of the sample size (up to 100), in agreement with what is commonly found for biological materials^69–72^. This suggests that due to the limitation of the tensile testing technique and the inherent residual stresses on silk, the presence of secondary structure differences in these fibres as well as the presence of handling-induced damages cannot be eliminated. This is valid especially for spider silk fibres, which display smaller dimensions compared to the others and thus are more challenging to handle. Based on this we conclude that the mechanical properties of silk materials and the natural variability of the population are significantly represented by testing at least 10 fibres. This could be also confirmed by Slovin’s formula for a population of 200 to estimate the size of a sample with margin of error corresponding to one standard deviation^73^, which gives ~ 8 as sample size. Furthermore, we argue that results from tensile tests should be reported by not excluding any data from the data set (i.e. through data selection), which may lead to an overinterpretation of the results.

**Figure 3:**
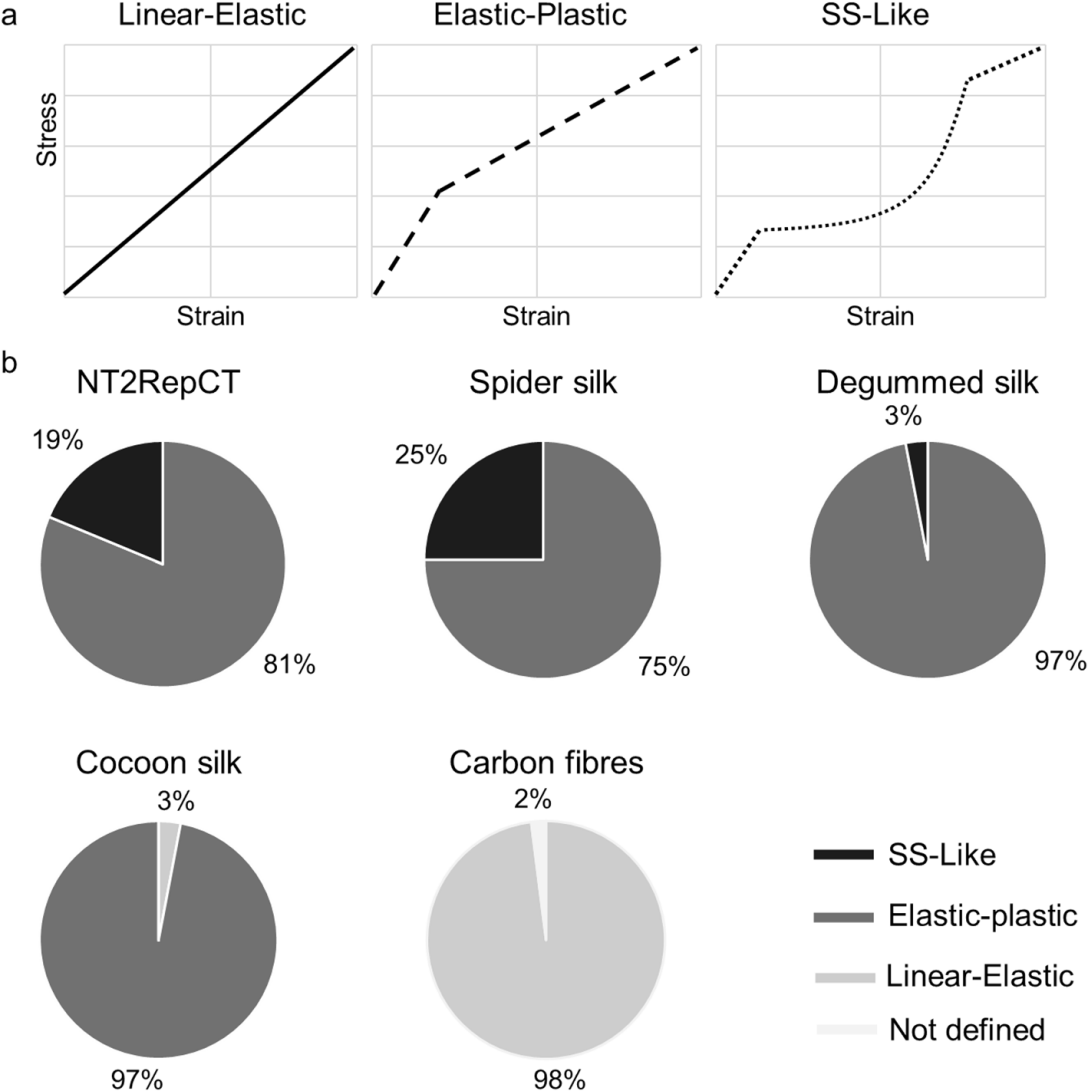
(**a**) Qualitative representation of typical stress–strain curves found in fibrous material. These models were used to classify the curves found in this work. In particular, for the SS-Like, we considered the curves with a pronounced non-linear phase (α > 1.5). (**b**) Graphics of the relative percentage of the qualitative shape distributions of the eng. stress–strain curves found in this work. For NT2RepCT, native spider silk, and degummed silk α was never higher than 3.

Since we obtained the shapes of 200 stress–strain curves together with the mechanical properties, we next assessed the typical elastic–plastic behaviour of the fibres. We divided the shape of curves into four groups: linear-elastic, elastic–plastic, theoretical Spider Silk like (SS-Like, Fig. 3a), and not defined. The linear-elastic is a stress–strain curve that displays a pure linear elastic behaviour till fracture. The elastic–plastic curve is defined by a linear elastic regime to the yield point, followed by a plastic deformation with a lower slope. The SS-like shape is the theoretical stress–strain curve described for spider silk (derived from atomistic models^42,74,75^), in which the first linear elastic part is followed, after the yield point, by a stiffening regime defined by the exponent *α* (see Eq. 1), which is here considered to be > 1.5. In this scenario, the true stress and true strain are not considered, as defined in^76^ (i.e. *ln* $(1+ \epsilon)$ for the true strain, and $s(1+ \epsilon)$ for the true stress), which are otherwise commonly used (e.g. ^8,77^), because it will mathematically affect the shape of the curve.

The majority of the carbon fibre specimens (98%) displayed the typical linear elastic behaviour (Figs. 3b and S6e), with very few examples of distorted curves (which were here classified “not defined”, which could mean that they were not physical). Possibly such curves may originate from fibres that were damaged during specimen preparation. The same can be said for cocoon silk fibre stress–strain curve, in which only a relatively small percentage (3%) presented a pure linear elastic behaviour (Figs. 3b and S6d), and prematurely broke. We attribute the main cause of these observations to specimen damage. The remaining 97% of the fibres, both for cocoon and degummed silk, displayed the well-known elastic–plastic behaviour typical of *B. mori* silk fibres. However, in degummed silk also a small percentage (3%) of SS-like stress–strain curves were found (Figs. 3b and S6c). The SS-like stress–strain curve is not unique to spider silk and it has been found also in fibres from non-mulberry silkworm, which seems to display a more homogeneous shape of such curves^44,45^. In the previously mentioned works^42,74,75^ it is proclaimed that the exponential stiffening is due to the gradual loading of the β-sheets while the amorphous regions are unfolding under the tensile load.. Thus, we believe that the degumming process, and in particular the boiling procedure, could affect the secondary structure of silk fibres and potentially induce a different mechanical behaviour^36,78^. Sericin remnants are less likely to cause this difference since no sericin can be found on the fibres that undergo degumming^36^.

Interestingly, although SS-like behaviour is usually considered to be the standard for spider silk fibres, in this work we found that only a relatively small fraction of curves displayed such behaviour (19% for NT2RepCT and 25% for native spider silk, Figs. 3b and S6a,b). In particular, in both the types of fibres none presented a characteristic non-linear stiffening exponent *α* > 3. Indeed, it seems that the majority of spider silks (artificial and native) fibres display an elastic–plastic behaviour, in which the plastic parts can be defined with 1 < *α* < 1.5. This aspect has been investigated by calculating the *α* parameter for different species (Table 1)^3,25,26^. For all the species considered, orb weavers or not, *α* < 1.5. It must be said that an ulterior stiffening of the fibres may be caused, under natural conditions, by the well-known supercontraction effects^24,79^, which can induce up to 100 MPa prestress in the natural orb radial webs fibres^80–83^, and thus the silk experiences further stiffening. Also, the mass of the spider can itself change the prestress conditions on the silk. This apart, we believe that the previously mentioned models could be further improved with *α* measured experimentally for the considered species.

**Table 1:** Values of the α parameter for the different species of spiders, associated with the type of web and silk they produce, obtained by fitting the stress-strain curves between 0.1 and 0.25 level of strain.

Species	Type of web	Type of silk	α measured	Reference
/	/	NT2RepCT	1.2 ± 0.1	This work
*Steatoda triangulosa*	Tangle web	Major ampullate	1.5 ± 0.5	This work, and Greco and Pugno^14^
*Steatoda paykulliana*	Tangle web	Major ampullate	1.2 ± 0.2	Greco and Pugno^14^
*Cupiennius salei*	None	Major ampullate	1.3 ± 0.2	Greco et al.^26^
*Nuctenea umbratica*	Orb web	Major ampullate	1.4 ± 0.3	Greco and Pugno^25^
*Araneus diadematus*	Orb web	Major ampullate	1.3 ± 0.1	Greco and Pugno^25^
*Zygiella x-notata*	Orb web	Major ampullate	1.4 ± 0.4	Greco and Pugno^25^

## Conclusions

Tensile testing is the most common technique to measure fibres’ mechanical properties, silk included. In the last decades, many concepts, hypothesis and theories on silk fibres have been introduced based on such tests. The number of tensile tests per fibre typology has varied substantially (ranging from a few units to sets of ten), and low variability (low standard deviation compared to the average) has been commonly reported.

In this study, we show that different silks fibres and a commercial type of carbon fibres have high variability despite the number of specimens that has been tested (10–70%). This study is important to the silk community to the extent to which it shows that 10 fibres could be sufficiently representative of the population and that a low variability in a mechanical properties data set is likely to be due to data selection. Moreover, we show that the nonlinear stiffening behaviour of spider major ampullate silk is commonly described with 1 < *α* < 1.5 and never > 3. This is important for further simulations analysis that will aim to describe the mechanical behaviour of structures composed also of major ampullate silk.

## Materials and methods

### Artificial spider silk

The spinning of the NT2RepCT fibres has been done based on the protocol described in Schmuck et al.^22^ in which the details on the protein purification and expression can be found. The protein solution with a concentration of 300 mg/ml was poured into a syringe. A neMESYS low pressure (290 N) syringe pump (Cetoni, Korbußen, Germany) was used to extrude the protein into the collection bath (700 mM Acetate buffer, pH 5 at a flow rate of 17 μl/min) through of a glass capillary of around 60 μm of diameter. The fibres were then collected in air on frames attached to a rotating (29 cm/s) wheel (diameter 11 cm) placed at the end of an 80 cm spinning bath.

### Natural silk and carbon fibres

Natural spider (major ampullate) silk was forcibly silk from a *Steatoda triangulosa* at ~ 1 cm/s. The spider was kept in captivity in the laboratory and fed and watered weekly. *Bombyx mori* cocoons were kindly provided by Chul Thai Silk Co., Ltd. (Petchaboon province, Thailand). The silk threads obtained from the cocoons were taken from adjacent filaments in the outer region of the cocoon. The degumming process follows an established protocol^1^. The used carbon fibres were Carbon C T24-5.0/270-E100 (SGL).

### Tensile tests

200 fibres per type were mounted on paper frames as described in Greco et al.^3^ and they were tested a few days later to minimize residual stresses. In particular, silk fibres were glued using double-sided tape on a paper frame with a squared window of 1 cm in side. The carbon fibres were glued, with the support of double side tape and super glue, on the same paper frame. Fibres were tested at five different sample sizes, namely 10, 20, 30, 40, and 100. All the experiments were performed in a room with controlled environmental conditions, in which the fibres were also stored with a relative humidity 20–35% and temperature 18–21 °C. The tensile tests were performed using an Agilent Technology nanotensile UTM T150. The strain rate was 1%/s. The 1% is relative to the initial gauge length of the fibre (~ 1 cm). The declared sensitivity of the machine is 1 nN for the load and 0.1 nm for the displacement. For each fibre, the diameter was measured utilizing an optical microscope (Olympus BX61) in at least three points for each fibre and then calculating the average. This was used to obtain the engineering stress assuming the cross-section as circular, which was plotted versus engineering strain. Since the NT2RepCT fibres do not have a circular cross-section, the considered diameter is depicted in Fig. S7. The Young’s modulus has been obtained from the slope of the line fit in initial linear part of the curves till 1% of strain, the strength and strain at break as the last point’s (before fracture) coordinates that also correspond to the maximal values. Toughness modulus was calculated from the area under the engineering stress–strain curve. For each sample size, the mean (*μ*) and the standard deviation (*σ*) were computed. Subsequentially, we computed the relative standard deviation as

2 $$\sigma_{rel} = \frac{{\sigma_{s} }}{{\mu_{s} }}$$

This was done to have a non-dimensional comparable unit. From the nonlinear *α* parameter, we used the protocol defined in Greco et al.^14^, i.e. fitting the curve with Eq. 1 and then obtaining the parameter *α*. In this sense, we also used previously published data (in open access) for other species, and in particular from their stress–strain curves^3,25,26^.

### Sample size calculation

To estimate the size of a sample, given a population with size N and unknown average and standard deviation, one can use Slovin’s formula^73^

3 $$n = \frac{N}{{1 + Ne^{2} }}$$

where *e* is the margin of error.

## Supplementary Information

Supplementary Information.

## Data Availability

All the data are included in the manuscript and are available in different formats upon request to the corresponding authors. The datasets generated and/or analysed during the current study are not publicly available since they are currently being used for further experiments, but are available from the corresponding author on request.
