# Supplementary material for: Artificial and natural silk materials have high mechanical property variability regardless of sample size
Source: Sci Rep. 2022 Mar 3;12:3507. doi: 10.1038/s41598-022-07212-5 (PMC8894418; doi:10.1038/s41598-022-07212-5)
Supplement: Supplementary file 1 — Supplementary Information. [file 41598_2022_7212_MOESM1_ESM.docx]

**Supplementary information**

Artificial and natural silk materials have high mechanical property variability regardless of sample size

Gabriele Greco ^a,b^, Hamideh Mirbaha ^a^, Benjamin Schmuck^b,c^, Anna Rising^b,c^ and Nicola M. Pugno^a,d*^

*^a^ Laboratory for Bioinspired, Bionic, Nano, Meta, Materials & Mechanics, Department of Civil, Environmental and Mechanical Engineering, University of Trento, Via Mesiano, 77, 38123 Trento, Italy*

*^b^ Department of Anatomy, Physiology and Biochemistry, Swedish University of Agricultural Sciences, Uppsala, Sweden*

*^c^ Department of Biosciences and Nutrition, Karolinska Institutet, Neo, 141 86 Huddinge, Sweden*

*^d^School of Engineering and Materials Science, Queen Mary University of London, Mile End Road, London E1 4NS, UK*

* Corresponding author: [gabriele.greco-2@unitn.it](mailto:gabriele.greco-2@unitn.it); [nicola.pugno@unitn.it](mailto:nicola.pugno@unitn.it);

Keywords: statistics; standard deviation; spider silk; silkworm silk; carbon fibres; nonlinear stiffening; *Bombyx mori*;


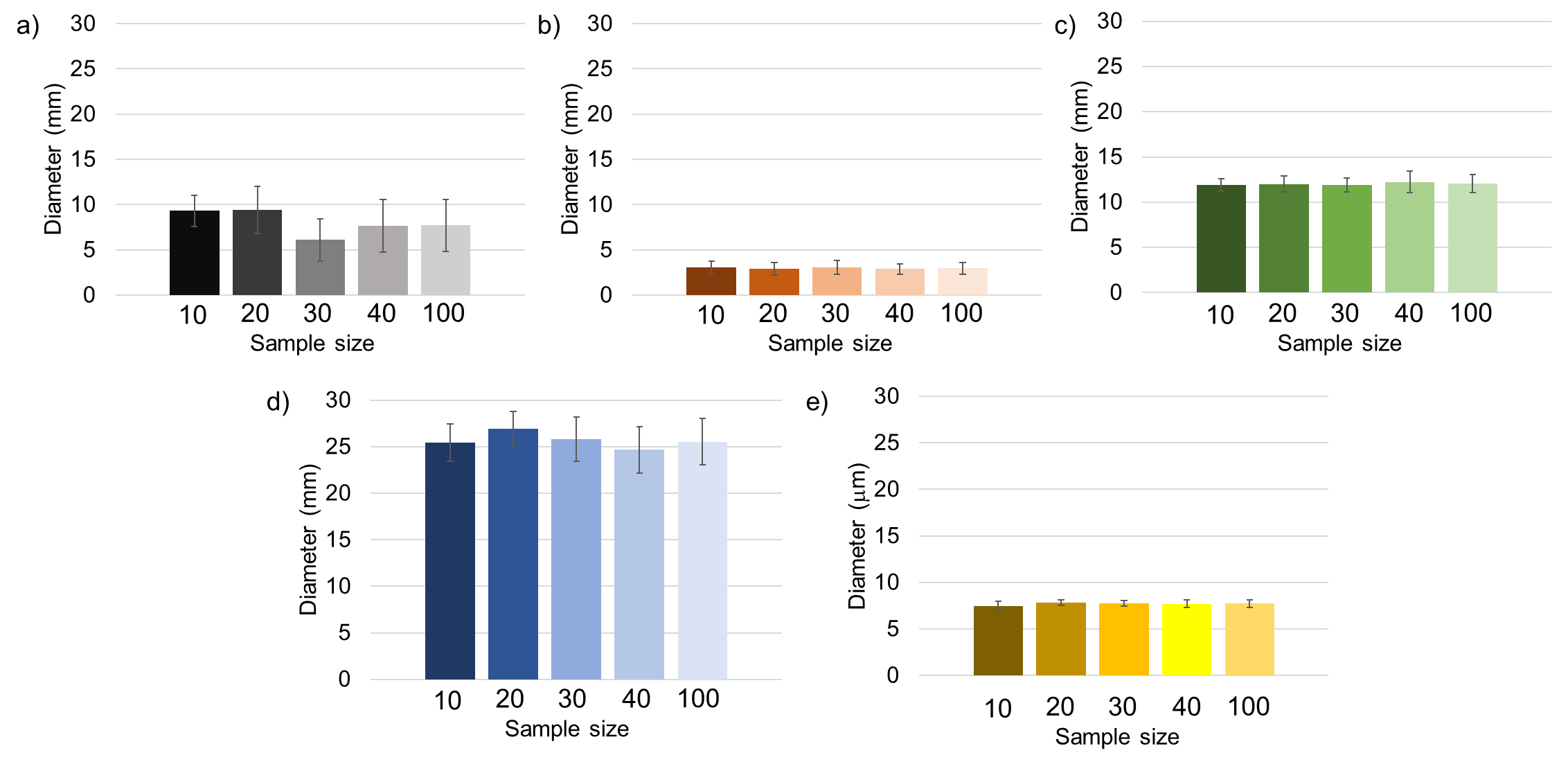


Figure S1: Diameter’s values vs the sample size for the different tested fibres: a) NT2repCT fibres, b) S. triangulosa fibres, c) degummed Bombyx mori fibres, d) cocoon Bombyx mori fibres, and e) commercial carbon fibres.


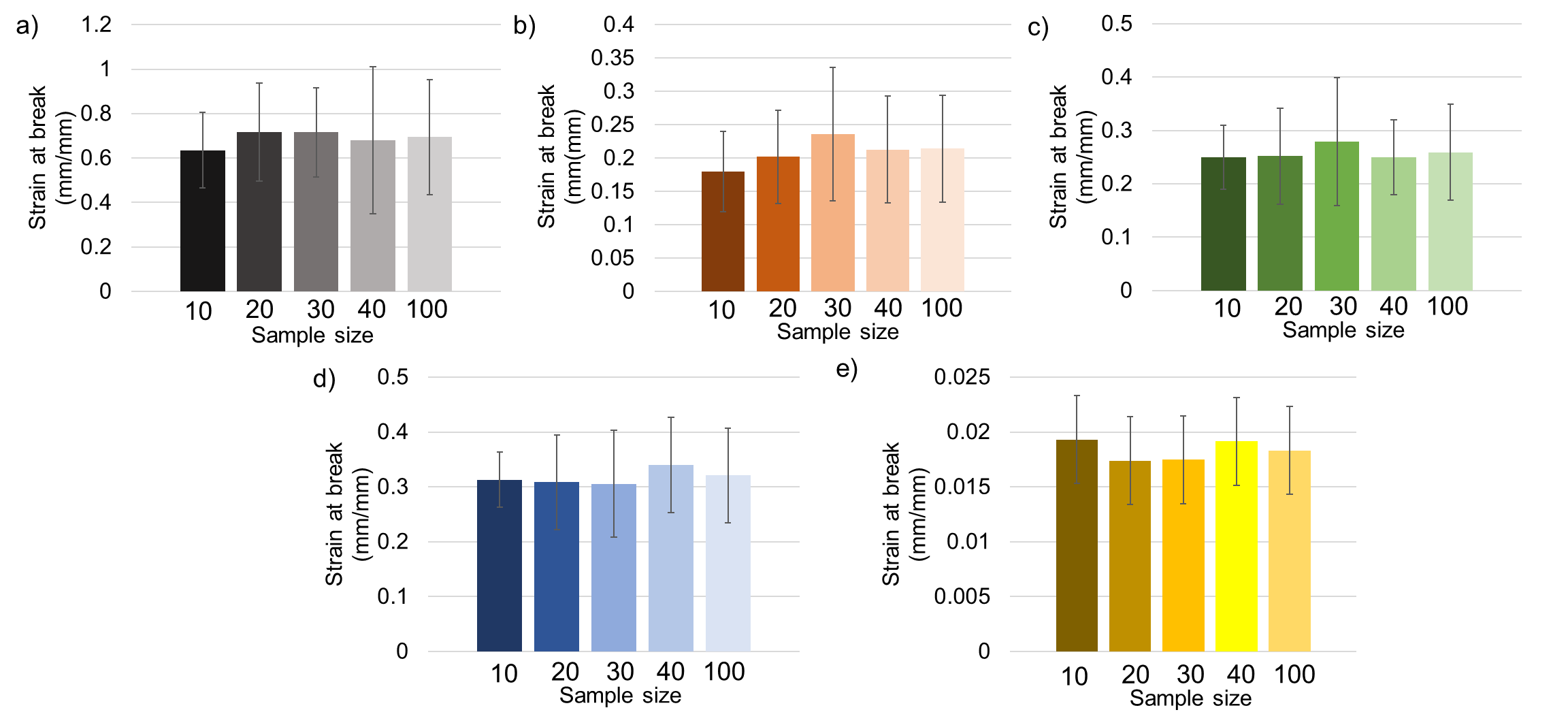


Figure S2: Strain at break’s values vs the sample size for the different tested fibres: a) NT2repCT fibres, b) S. triangulosa fibres, c) degummed Bombyx mori fibres, d) cocoon Bombyx mori fibres, and e) commercial carbon fibres.


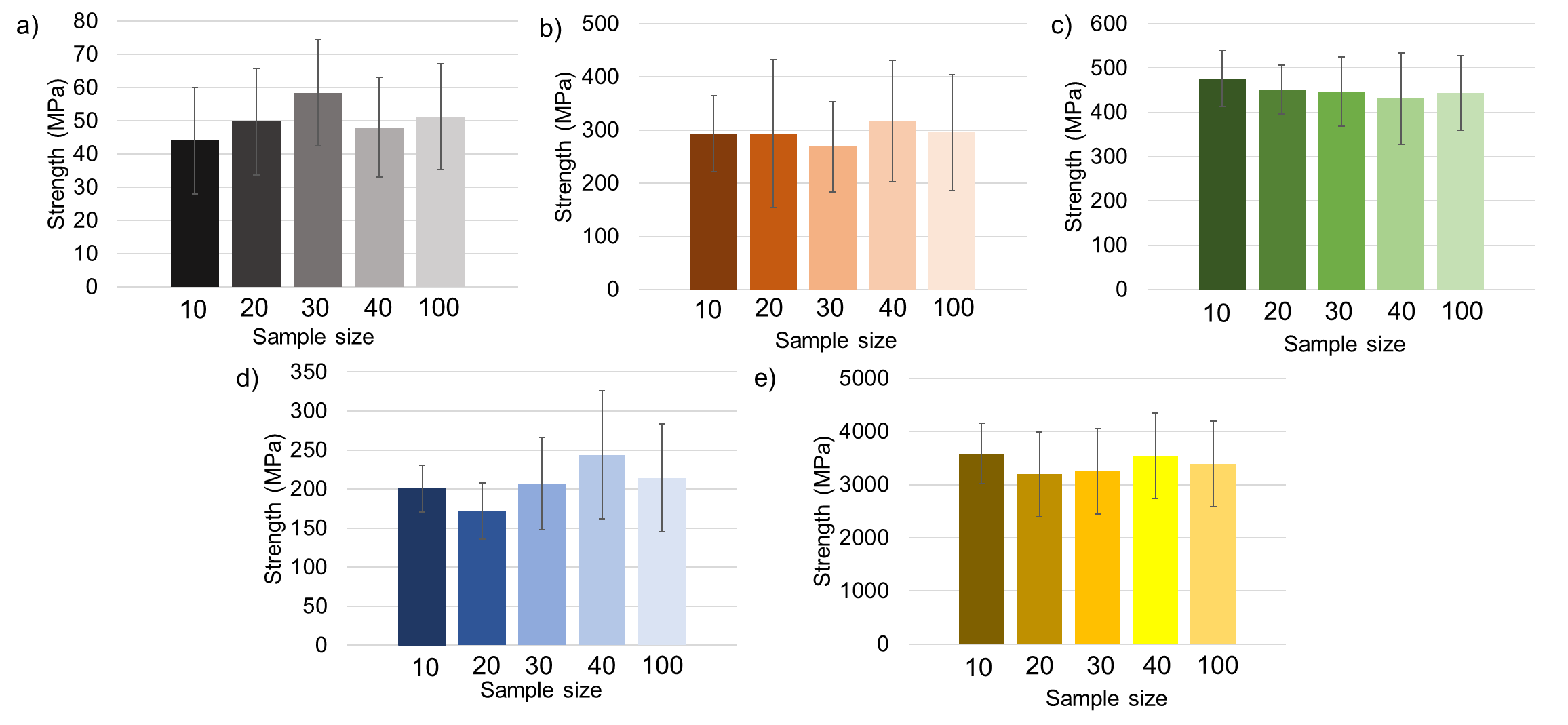


Figure S3: Strength’s values vs the sample size for the different tested fibres: a) NT2repCT fibres, b) S. triangulosa fibres, c) degummed Bombyx mori fibres, d) cocoon Bombyx mori fibres, and e) commercial carbon fibres.


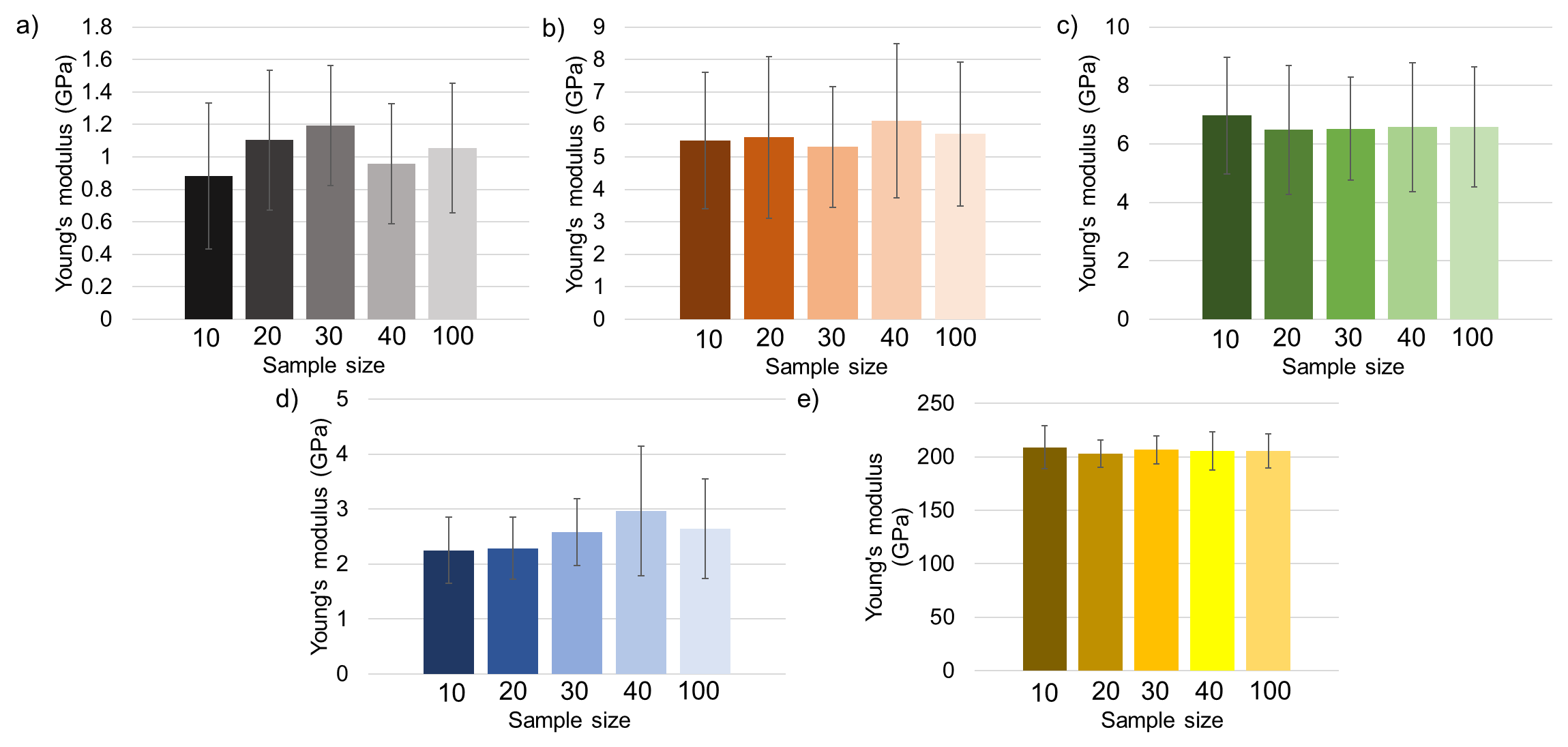


Figure S4: Young’s modulus values vs the sample size for the different tested fibres: a) NT2repCT fibres, b) S. triangulosa fibres, c) degummed Bombyx mori fibres, d) cocoon Bombyx mori fibres, and e) commercial carbon fibres.


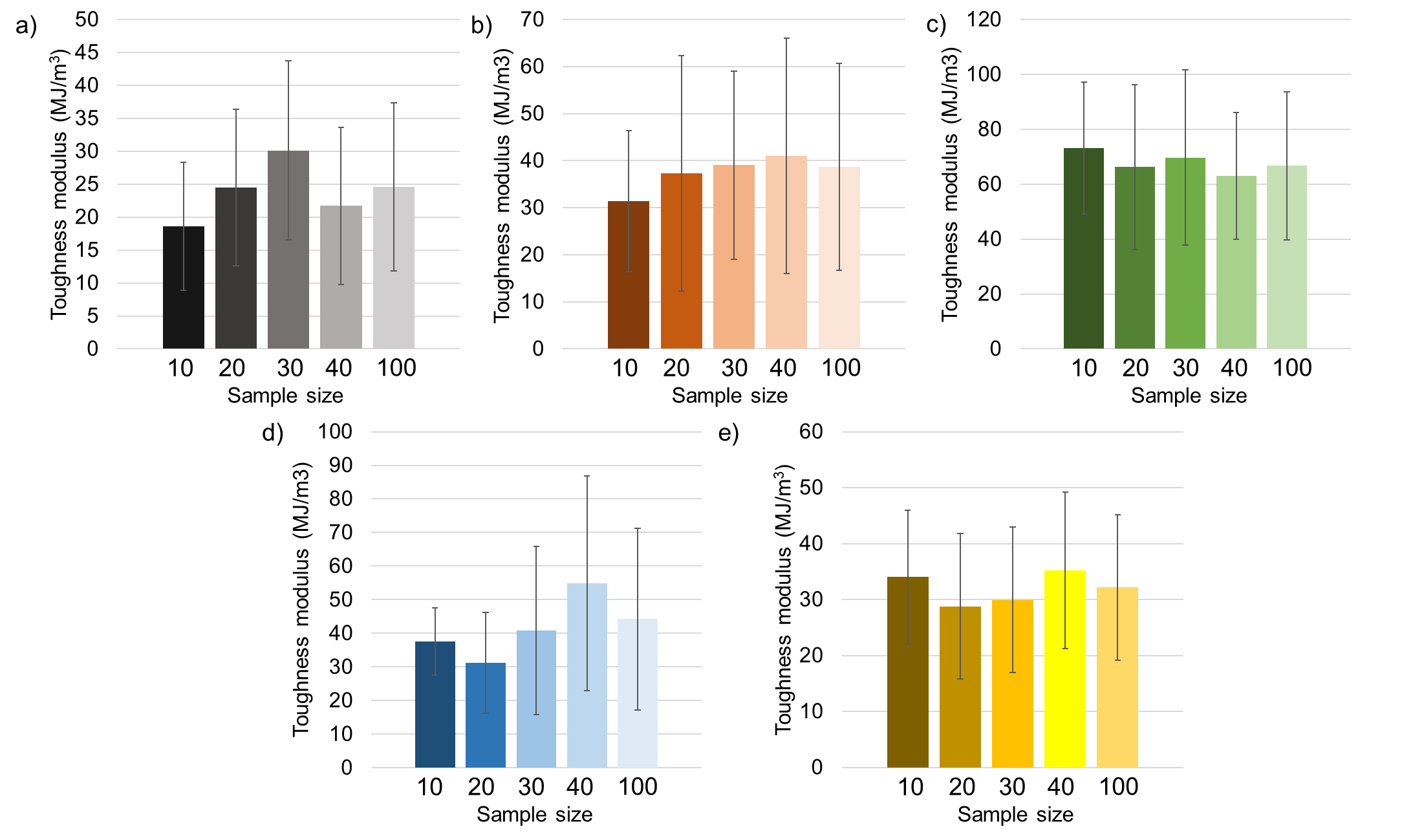


Figure S5: Toughness modulus values vs the sample size for the different tested fibres: a) NT2repCT fibres, b) S. triangulosa fibres, c) degummed Bombyx mori fibres, d) cocoon Bombyx mori fibres, and e) commercial carbon fibres.


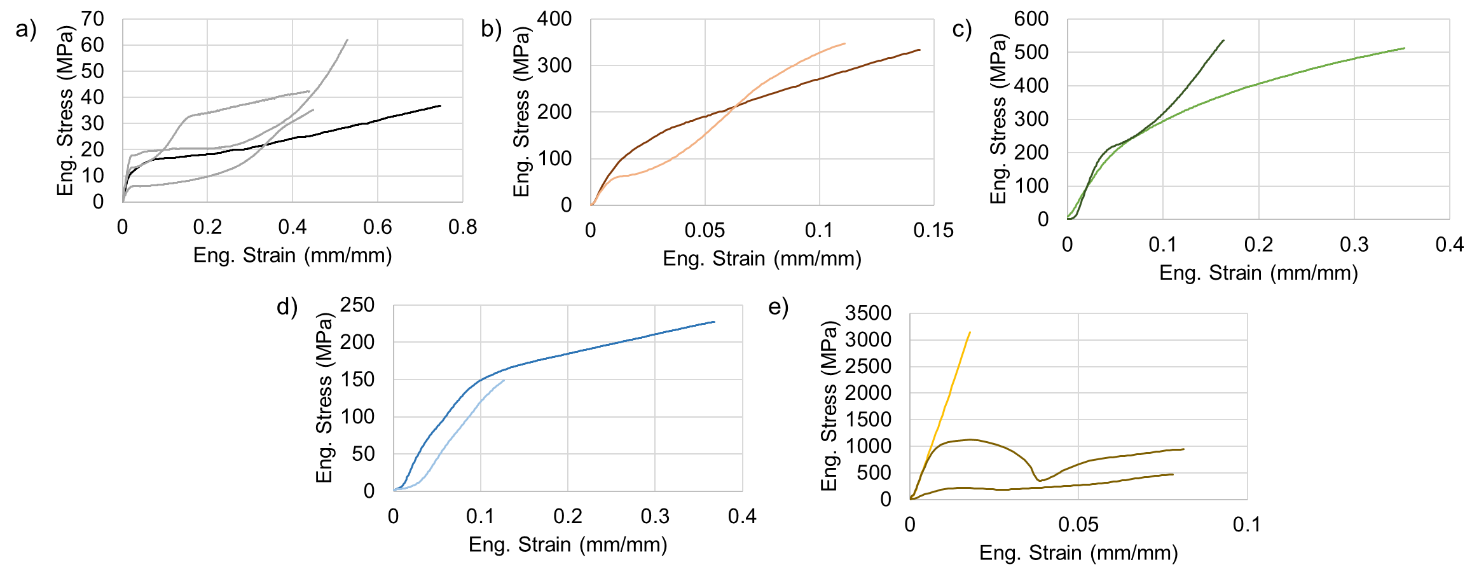


Figure S6: Representative selected eng. stress and strain curves (with different shapes) from the fibres dataset that were tested. a) NT2repCT fibres, b) S. triangulosa fibres, c) degummed Bombyx mori fibres, d) cocoon Bombyx mori fibres, and e) commercial carbon fibres, in which are also depicted two not-defined Eng. stress strain curves.


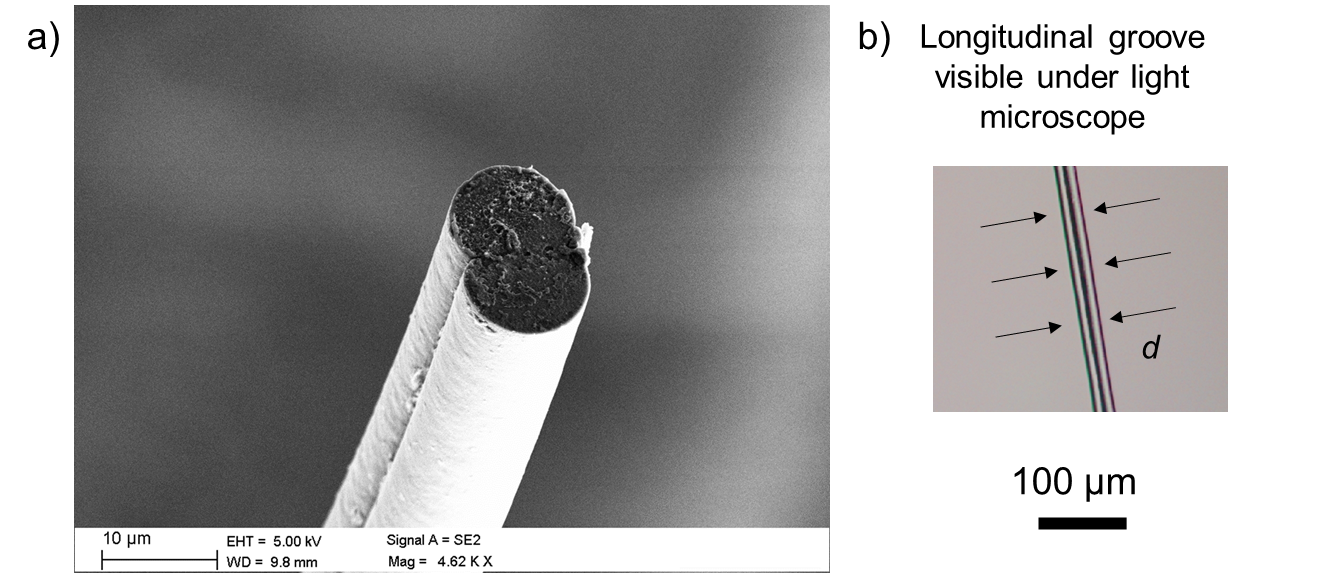


Figure S7: a) SEM image of a NT2RepCT fibre studied in this work. The cross section of the fibre is not circular with a longitudinal groove on one side. Adapted from Schmuck et al., Mater. Today, 2021. b) The diameter (d) was measure with the light microscope as depicted here, considering the side of the fibre where the longitudinal groove was visible. In this way, the mechanical properties strength, Young’s modulus, and toughness modulus of the NT2RepCT fibres are underestimated.
